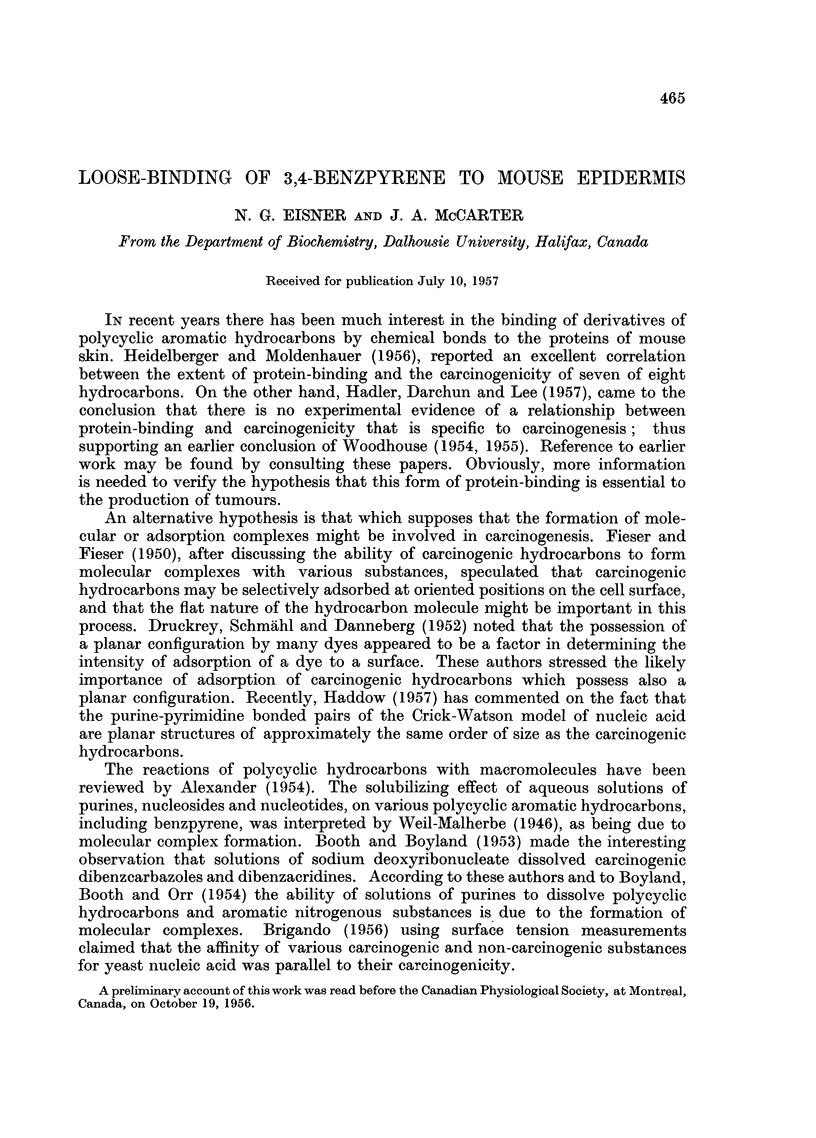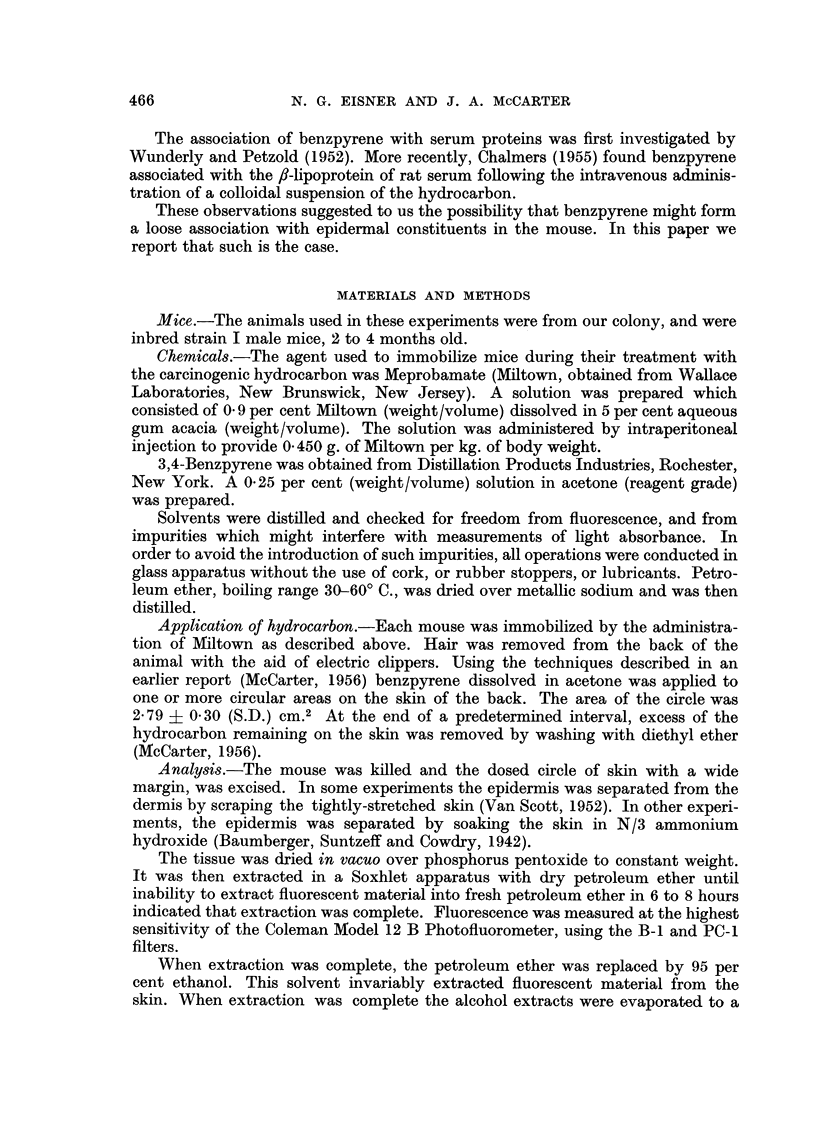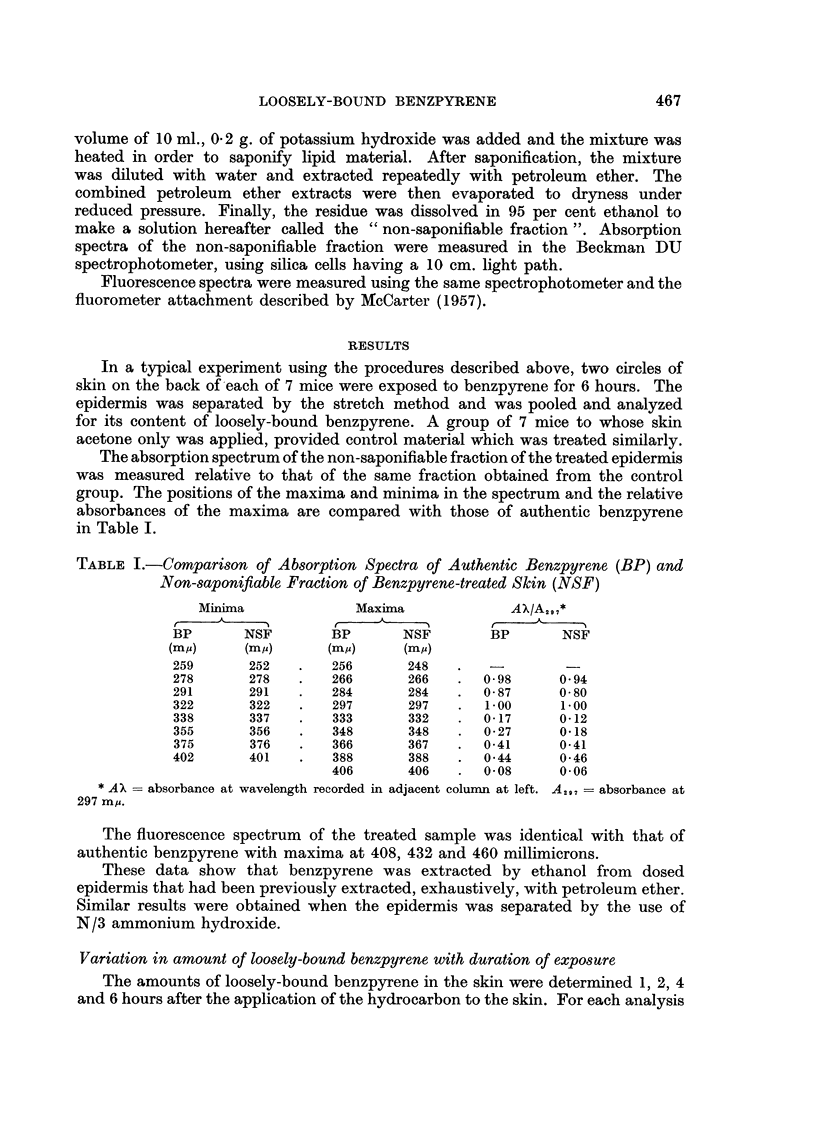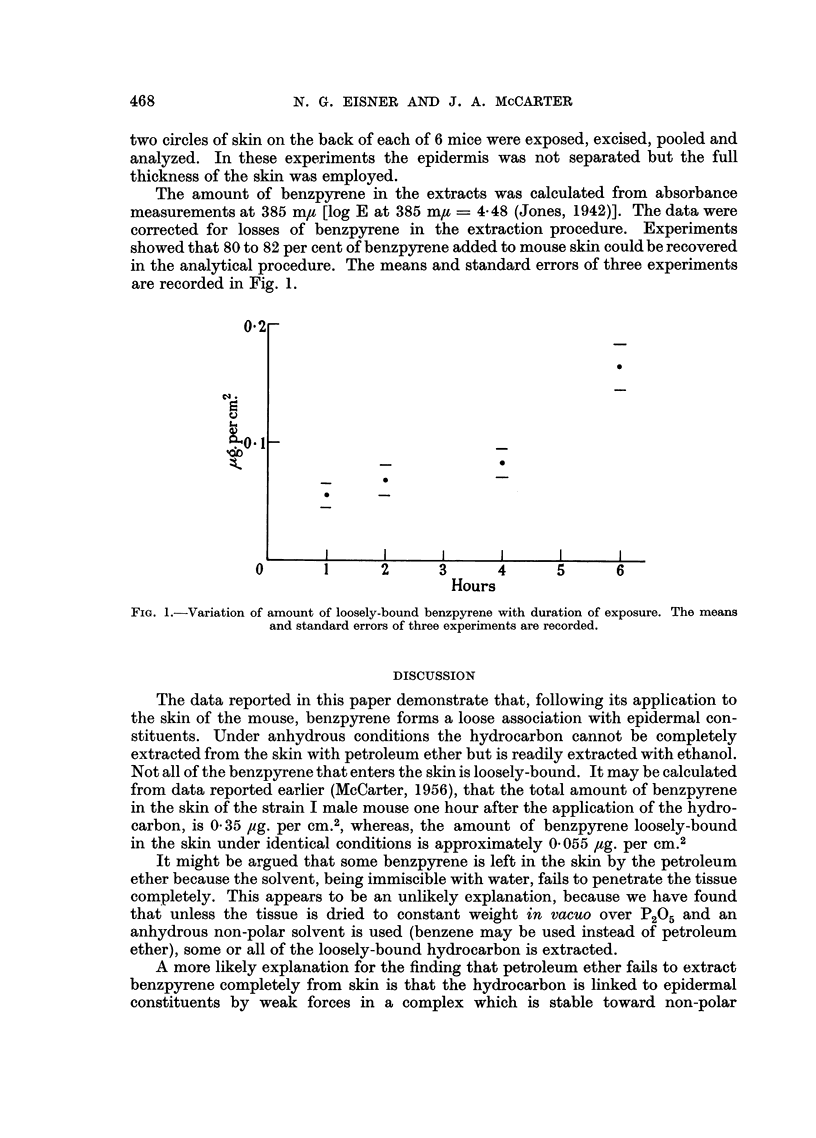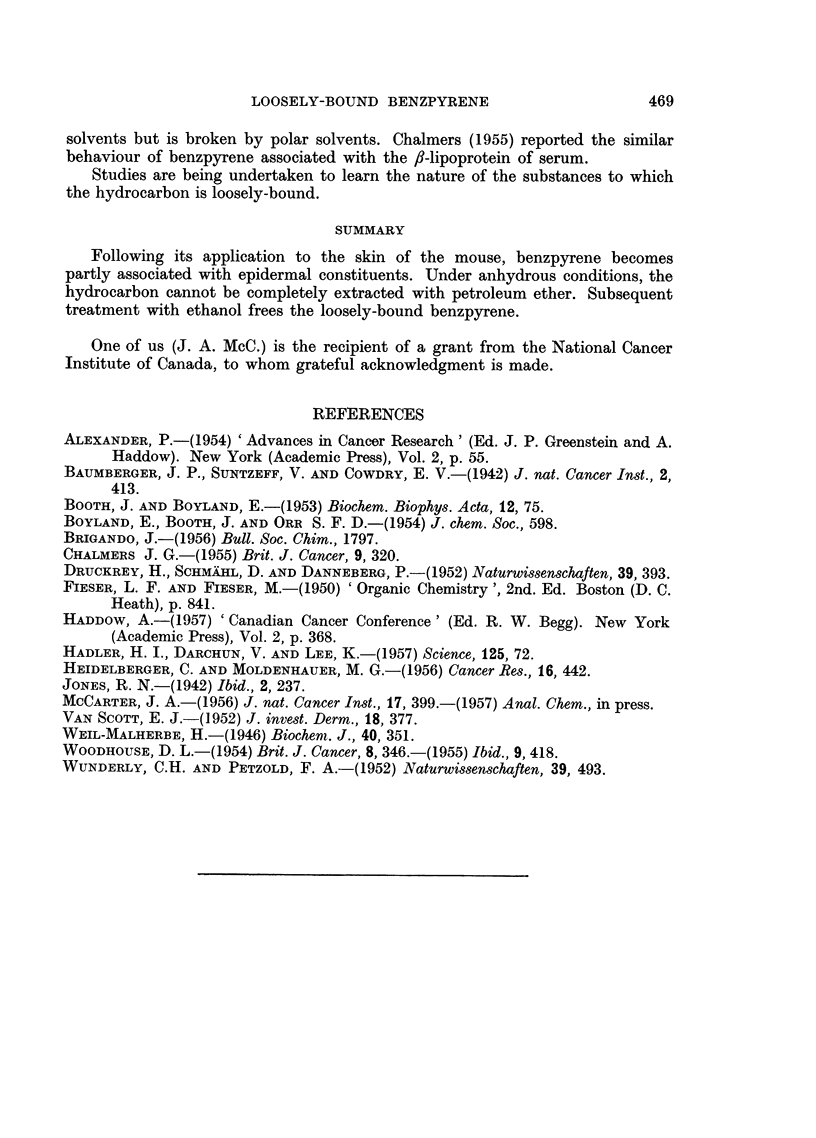# Loose-Binding of 3,4-Benzpyrene to Mouse Epidermis

**DOI:** 10.1038/bjc.1957.54

**Published:** 1957-09

**Authors:** N. G. Eisner, J. A. McCarter


### LOOSE-BINDING OF 3,4-BENZPYRENE TO MOUSE EPIDERMIS

N. G. EISNER AND J. A. McCARTER

*From the Department of Biochemistry, Dalhousie University, Halifax, Canada*

Received for publication July 10, 1957

IN recent years there has been much interest in the binding of derivatives of polycyclic aromatic hydrocarbons by chemical bonds to the proteins of mouse skin. Heidelberger and Moldenhauer (1956), reported an excellent correlation between the extent of protein-binding and the carcinogenicity of seven of eight hydrocarbons. On the other hand, Hadler, Darchun and Lee (1957), came to the conclusion that there is no experimental evidence of a relationship between protein-binding and carcinogenicity that is specific to carcinogenesis; thus supporting an earlier conclusion of Woodhouse (1954, 1955). Reference to earlier work may be found by consulting these papers. Obviously, more information is needed to verify the hypothesis that this form of protein-binding is essential to the production of tumours.

An alternative hypothesis is that which supposes that the formation of molecular or adsorption complexes might be involved in carcinogenesis. Fieser and Fieser (1950), after discussing the ability of carcinogenic hydrocarbons to form molecular complexes with various substances, speculated that carcinogenic hydrocarbons may be selectively adsorbed at oriented positions on the cell surface, and that the flat nature of the hydrocarbon molecule might be important in this process. Druckrey, Schmähl and Danneberg (1952) noted that the possession of a planar configuration by many dyes appeared to be a factor in determining the intensity of adsorption of a dye to a surface. These authors stressed the likely importance of adsorption of carcinogenic hydrocarbons which possess also a planar configuration. Recently, Haddow (1957) has commented on the fact that the purine-pyrimidine bonded pairs of the Crick-Watson model of nucleic acid are planar structures of approximately the same order of size as the carcinogenic hydrocarbons.

The reactions of polycyclic hydrocarbons with macromolecules have been reviewed by Alexander (1954). The solubilizing effect of aqueous solutions of purines, nucleosides and nucleotides, on various polycyclic aromatic hydrocarbons, including benzpyrene, was interpreted by Weil-Malherbe (1946), as being due to molecular complex formation. Booth and Boyland (1953) made the interesting observation that solutions of sodium deoxyribonucleate dissolved carcinogenic dibenzcarbazoles and dibenzacridines. According to these authors and to Boyland, Booth and Orr (1954) the ability of solutions of purines to dissolve polycyclic hydrocarbons and aromatic nitrogenous substances is due to the formation of molecular complexes. Brigando (1956) using surface tension measurements claimed that the affinity of various carcinogenic and non-carcinogenic substances for yeast nucleic acid was parallel to their carcinogenicity.

A preliminary account of this work was read before the Canadian Physiological Society, at Montreal, Canada, on October 19, 1956.

The association of benzpyrene with serum proteins was first investigated by Wunderly and Petzold (1952). More recently, Chalmers (1955) found benzpyrene associated with the $\beta$-lipoprotein of rat serum following the intravenous administration of a colloidal suspension of the hydrocarbon.

These observations suggested to us the possibility that benzpyrene might form a loose association with epidermal constituents in the mouse. In this paper we report that such is the case.

### MATERIALS AND METHODS

*Mice.*—The animals used in these experiments were from our colony, and were inbred strain I male mice, 2 to 4 months old.

*Chemicals.*—The agent used to immobilize mice during their treatment with the carcinogenic hydrocarbon was Meprobamate (Miltown, obtained from Wallace Laboratories, New Brunswick, New Jersey). A solution was prepared which consisted of 0·9 per cent Miltown (weight/volume) dissolved in 5 per cent aqueous gum acacia (weight/volume). The solution was administered by intraperitoneal injection to provide 0·450 g. of Miltown per kg. of body weight.

3,4-Benzpyrene was obtained from Distillation Products Industries, Rochester, New York. A 0·25 per cent (weight/volume) solution in acetone (reagent grade) was prepared.

Solvents were distilled and checked for freedom from fluorescence, and from impurities which might interfere with measurements of light absorbance. In order to avoid the introduction of such impurities, all operations were conducted in glass apparatus without the use of cork, or rubber stoppers, or lubricants. Petroleum ether, boiling range 30–60° C., was dried over metallic sodium and was then distilled.

*Application of hydrocarbon.*—Each mouse was immobilized by the administration of Miltown as described above. Hair was removed from the back of the animal with the aid of electric clippers. Using the techniques described in an earlier report (McCarter, 1956) benzpyrene dissolved in acetone was applied to one or more circular areas on the skin of the back. The area of the circle was $2{\cdot}79 \pm 0{\cdot}30$ (S.D.) cm.$^2$ At the end of a predetermined interval, excess of the hydrocarbon remaining on the skin was removed by washing with diethyl ether (McCarter, 1956).

*Analysis.*—The mouse was killed and the dosed circle of skin with a wide margin, was excised. In some experiments the epidermis was separated from the dermis by scraping the tightly-stretched skin (Van Scott, 1952). In other experiments, the epidermis was separated by soaking the skin in N/3 ammonium hydroxide (Baumberger, Suntzeff and Cowdry, 1942).

The tissue was dried *in vacuo* over phosphorus pentoxide to constant weight. It was then extracted in a Soxhlet apparatus with dry petroleum ether until inability to extract fluorescent material into fresh petroleum ether in 6 to 8 hours indicated that extraction was complete. Fluorescence was measured at the highest sensitivity of the Coleman Model 12 B Photofluorometer, using the B-1 and PC-1 filters.

When extraction was complete, the petroleum ether was replaced by 95 per cent ethanol. This solvent invariably extracted fluorescent material from the skin. When extraction was complete the alcohol extracts were evaporated to a volume of 10 ml., 0·2 g. of potassium hydroxide was added and the mixture was heated in order to saponify lipid material. After saponification, the mixture was diluted with water and extracted repeatedly with petroleum ether. The combined petroleum ether extracts were then evaporated to dryness under reduced pressure. Finally, the residue was dissolved in 95 per cent ethanol to make a solution hereafter called the " non-saponifiable fraction ". Absorption spectra of the non-saponifiable fraction were measured in the Beckman DU spectrophotometer, using silica cells having a 10 cm. light path.

Fluorescence spectra were measured using the same spectrophotometer and the fluorometer attachment described by McCarter (1957).

### RESULTS

In a typical experiment using the procedures described above, two circles of skin on the back of each of 7 mice were exposed to benzpyrene for 6 hours. The epidermis was separated by the stretch method and was pooled and analyzed for its content of loosely-bound benzpyrene. A group of 7 mice to whose skin acetone only was applied, provided control material which was treated similarly.

The absorption spectrum of the non-saponifiable fraction of the treated epidermis was measured relative to that of the same fraction obtained from the control group. The positions of the maxima and minima in the spectrum and the relative absorbances of the maxima are compared with those of authentic benzpyrene in Table I.

TABLE I.—*Comparison of Absorption Spectra of Authentic Benzpyrene (BP) and Non-saponifiable Fraction of Benzpyrene-treated Skin (NSF)*

| Minima | | Maxima | | $A\lambda/A_{297}$* | |
|---|---|---|---|---|---|
| BP (mμ) | NSF (mμ) | BP (mμ) | NSF (mμ) | BP | NSF |
| 259 | 252 | 256 | 248 | — | — |
| 278 | 278 | 266 | 266 | 0·98 | 0·94 |
| 291 | 291 | 284 | 284 | 0·87 | 0·80 |
| 322 | 322 | 297 | 297 | 1·00 | 1·00 |
| 338 | 337 | 333 | 332 | 0·17 | 0·12 |
| 355 | 356 | 348 | 348 | 0·27 | 0·18 |
| 375 | 376 | 366 | 367 | 0·41 | 0·41 |
| 402 | 401 | 388 | 388 | 0·44 | 0·46 |
| | | 406 | 406 | 0·08 | 0·06 |

* $A\lambda$ = absorbance at wavelength recorded in adjacent column at left. $A_{297}$ = absorbance at 297 mμ.

The fluorescence spectrum of the treated sample was identical with that of authentic benzpyrene with maxima at 408, 432 and 460 millimicrons.

These data show that benzpyrene was extracted by ethanol from dosed epidermis that had been previously extracted, exhaustively, with petroleum ether. Similar results were obtained when the epidermis was separated by the use of N/3 ammonium hydroxide.

### *Variation in amount of loosely-bound benzpyrene with duration of exposure*

The amounts of loosely-bound benzpyrene in the skin were determined 1, 2, 4 and 6 hours after the application of the hydrocarbon to the skin. For each analysis two circles of skin on the back of each of 6 mice were exposed, excised, pooled and analyzed. In these experiments the epidermis was not separated but the full thickness of the skin was employed.

The amount of benzpyrene in the extracts was calculated from absorbance measurements at 385 m$\mu$ [log E at 385 m$\mu$ = 4·48 (Jones, 1942)]. The data were corrected for losses of benzpyrene in the extraction procedure. Experiments showed that 80 to 82 per cent of benzpyrene added to mouse skin could be recovered in the analytical procedure. The means and standard errors of three experiments are recorded in Fig. 1.

FIG. 1.—Variation of amount of loosely-bound benzpyrene with duration of exposure. The means and standard errors of three experiments are recorded.

### DISCUSSION

The data reported in this paper demonstrate that, following its application to the skin of the mouse, benzpyrene forms a loose association with epidermal constituents. Under anhydrous conditions the hydrocarbon cannot be completely extracted from the skin with petroleum ether but is readily extracted with ethanol. Not all of the benzpyrene that enters the skin is loosely-bound. It may be calculated from data reported earlier (McCarter, 1956), that the total amount of benzpyrene in the skin of the strain I male mouse one hour after the application of the hydrocarbon, is 0·35 $\mu$g. per cm.$^2$, whereas, the amount of benzpyrene loosely-bound in the skin under identical conditions is approximately 0·055 $\mu$g. per cm.$^2$

It might be argued that some benzpyrene is left in the skin by the petroleum ether because the solvent, being immiscible with water, fails to penetrate the tissue completely. This appears to be an unlikely explanation, because we have found that unless the tissue is dried to constant weight *in vacuo* over $P_2O_5$ and an anhydrous non-polar solvent is used (benzene may be used instead of petroleum ether), some or all of the loosely-bound hydrocarbon is extracted.

A more likely explanation for the finding that petroleum ether fails to extract benzpyrene completely from skin is that the hydrocarbon is linked to epidermal constituents by weak forces in a complex which is stable toward non-polar solvents but is broken by polar solvents. Chalmers (1955) reported the similar behaviour of benzpyrene associated with the $\beta$-lipoprotein of serum.

Studies are being undertaken to learn the nature of the substances to which the hydrocarbon is loosely-bound.

### SUMMARY

Following its application to the skin of the mouse, benzpyrene becomes partly associated with epidermal constituents. Under anhydrous conditions, the hydrocarbon cannot be completely extracted with petroleum ether. Subsequent treatment with ethanol frees the loosely-bound benzpyrene.

One of us (J. A. McC.) is the recipient of a grant from the National Cancer Institute of Canada, to whom grateful acknowledgment is made.

### REFERENCES

ALEXANDER, P.—(1954) ' Advances in Cancer Research ' (Ed. J. P. Greenstein and A. Haddow). New York (Academic Press), Vol. 2, p. 55.
BAUMBERGER, J. P., SUNTZEFF, V. AND COWDRY, E. V.—(1942) *J. nat. Cancer Inst.*, **2**, 413.
BOOTH, J. AND BOYLAND, E.—(1953) *Biochem. Biophys. Acta*, **12**, 75.
BOYLAND, E., BOOTH, J. AND ORR S. F. D.—(1954) *J. chem. Soc.*, 598.
BRIGANDO, J.—(1956) *Bull. Soc. Chim.*, 1797.
CHALMERS J. G.—(1955) *Brit. J. Cancer*, **9**, 320.
DRUCKREY, H., SCHMÄHL, D. AND DANNEBERG, P.—(1952) *Naturwissenschaften*, **39**, 393.
FIESER, L. F. AND FIESER, M.—(1950) ' Organic Chemistry ', 2nd. Ed. Boston (D. C. Heath), p. 841.
HADDOW, A.—(1957) ' Canadian Cancer Conference ' (Ed. R. W. Begg). New York (Academic Press), Vol. 2, p. 368.
HADLER, H. I., DARCHUN, V. AND LEE, K.—(1957) *Science*, **125**, 72.
HEIDELBERGER, C. AND MOLDENHAUER, M. G.—(1956) *Cancer Res.*, **16**, 442.
JONES, R. N.—(1942) *Ibid.*, **2**, 237.
McCARTER, J. A.—(1956) *J. nat. Cancer Inst.*, **17**, 399.—(1957) *Anal. Chem.*, in press.
VAN SCOTT, E. J.—(1952) *J. invest. Derm.*, **18**, 377.
WEIL-MALHERBE, H.—(1946) *Biochem. J.*, **40**, 351.
WOODHOUSE, D. L.—(1954) *Brit. J. Cancer*, **8**, 346.—(1955) *Ibid.*, **9**, 418.
WUNDERLY, C.H. AND PETZOLD, F. A.—(1952) *Naturwissenschaften*, **39**, 493.